# 2-(1*H*-1,2,3-Benzotriazol-1-yl)-*N*′-cyclo­pentyl­ideneacetohydrazide

**DOI:** 10.1107/S1600536808005631

**Published:** 2008-03-05

**Authors:** Ning-Ning Ji, Zhi-Qiang Shi

**Affiliations:** a Department of Chemistry, Taishan University, 271021 Taian, Shandong, People’s Republic of China; b Department of Materials Science and Chemical Engineering, Taishan University, 271021 Taian, Shandong, People’s Republic of China

## Abstract

The title compound, C_13_H_15_N_5_O, was synthesized by the reaction of 2-(1*H*-1,2,3-benzotriazol-1-yl)acetohydrazide with cyclo­penta­none. In the cyclopentane ring, two C atoms and their attached H atoms are disordered over two positions; the site occupancy factors are ca 0.63 and 0.37. In the crystal structure, mol­ecules are linked into infinite chains directed along the *b* axis by N—H⋯O hydrogen bonds. In addition, there are weak C—H⋯O and C—H⋯N hydrogen bonds, as well as C—H⋯π-ring inter­actions in the structure.

## Related literature

For related literature, see: Allen (2002 ▶); Allen *et al.* (1987 ▶); Garnovskii *et al.* (1993 ▶); Anderson *et al.* (1997 ▶); Müller *et al.* (2006 ▶); Musie *et al.* (2001 ▶); Xu *et al.* (2002 ▶); Ghosh *et al.* (2002 ▶); Shi *et al.* (2007 ▶); Yang (2006 ▶).
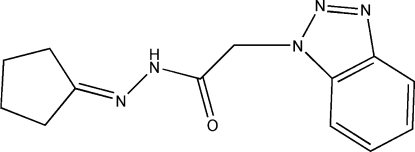


## Experimental

### 

#### Crystal data

C_13_H_15_N_5_O
*M* *_r_* = 257.30
Monoclinic, 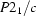

*a* = 11.926 (3) Å
*b* = 9.126 (2) Å
*c* = 12.095 (3) Å
β = 93.174 (5)°
*V* = 1314.4 (5) Å^3^
*Z* = 4
Mo *K*α radiation
μ = 0.09 mm^−1^
*T* = 295 (2) K
0.32 × 0.24 × 0.11 mm

#### Data collection

Bruker SMART CCD area-detector diffractometer
Absorption correction: multi-scan (*SADABS*; Sheldrick, 1996 ▶) *T*_min_ = 0.972, *T*_max_ = 0.990
6723 measured reflections
2323 independent reflections
1114 reflections with *I* > 2σ(*I*)
*R*_int_ = 0.065

#### Refinement

*R*[*F* ^2^ > 2σ(*F* ^2^)] = 0.059
*wR*(*F* ^2^) = 0.170
*S* = 1.01
2323 reflections
173 parameters
7 restraints
H-atom parameters constrained
Δρ_max_ = 0.17 e Å^−3^
Δρ_min_ = −0.26 e Å^−3^

### 

Data collection: *SMART* (Siemens, 1996 ▶); cell refinement: *SMART*; data reduction: *SAINT* (Siemens, 1996 ▶); program(s) used to solve structure: *SHELXS97* (Sheldrick, 2008 ▶); program(s) used to refine structure: *SHELXL97* (Sheldrick, 2008 ▶); molecular graphics: *SHELXTL* (Sheldrick, 2008 ▶); software used to prepare material for publication: *SHELXTL*.

## Supplementary Material

Crystal structure: contains datablocks global, I. DOI: 10.1107/S1600536808005631/fb2083sup1.cif

Structure factors: contains datablocks I. DOI: 10.1107/S1600536808005631/fb2083Isup2.hkl

Additional supplementary materials:  crystallographic information; 3D view; checkCIF report

## Figures and Tables

**Table 1:** Hydrogen-bond geometry (Å, °)

*D*—H⋯*A*	*D*—H	H⋯*A*	*D*⋯*A*	*D*—H⋯*A*
N4—H4⋯O1^i^	0.86	2.17	2.910 (3)	144
C7—H7*B*⋯O1^i^	0.97	2.58	3.435 (4)	147
C7—H7*B*⋯N5^i^	0.97	2.51	3.372 (4)	147
C13—H13*A*⋯*Cg*1^ii^	0.97	2.79	3.729 (4)	163
C12′—H12*C*⋯*Cg*2^ii^	0.97	2.99	3.820 (19)	145

Symmetry codes: (i) 
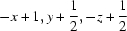
; (ii) 
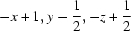
. *Cg*1 and *Cg*2 are the centroids of the N1,N2,N3,C1,C2 and C1–C6 rings, respectively.

**Fig. 1.**
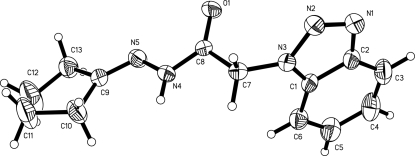
The molecular structure of the title compound with the displacement ellipsoids drawn at the 50% probability level.

**Fig. 2.**
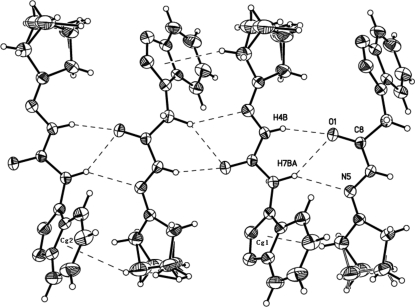
The crystal structure of the title compounds showing the infinite chains interconnected via the H—H···O hydrogen bonds. The dashed lines indicate the hydrogen bonds.

## supplementary crystallographic information

### Comment

Recently, a number of Schiff-bases have been investigated because of their interesting coordination chemistry (Garnovskii *et al.*, 1993; Musie *et al.*, 2001; Ghosh *et al.*, 2002; Shi *et al.*, 2007) as well as due to their importance in biological systems (Anderson *et al.*, 1997). The Schiff-bases containing the triazole group have attracted much attention because they exhibit potential bioactivities (Xu *et al.*, 2002). In order to search for new triazole compounds with higher bioactivity, the title compound was synthesized and its crystal structure determined (Fig. 1 and Fig. 2). The bond lengths and angles are in good agreement with the expected values (Allen *et al.*, 1987). In the crystal structure, the molecules are linked into infinite chains by the N—H···O hydrogen bonds. In addition, there are also present weak C—H···O and C—H···N hydrogen bonds as well as C—H···π-ring interactions (Tab. 1). *Cg*1 and *Cg*2 are the centroids pertinent to the rings N1\N2\N3\C1\C2 and C1\C2\···C6, respectively.

### Experimental

The title compound was synthesized by the reaction of 2-(1*H*-1,2,3-benzotriazol-1-yl)acetohydrazide (1 mmol, 191.2 mg) with cyclopentanone (1 mmol, 84.1 mg) in ethanol (25 ml). The mixture was refluxed at 338 K for 4 h until a clear solution occurred. After ten days, colourless block crystals with approx. size 0.3 × 0.2 × 0.1 mm suitable for X-ray diffraction study were obtained. Yield, 257.3 mg, 87%. m. p. 490–492 K.

Analysis calculated for C_13_H_15_N_5_O: C 60.69, H 5.88, N 27.22%; found: C 60.66, H 5.82, N 27.17%.

### Refinement

The majority of the H atoms could have been determined in the difference Fourier maps with exception of the disordered atoms C11, C11', C12 and C12'. During the refinement the H atoms were situated into idealized positions, constrained and refined as riding atoms. The constraints: C_aryl_—H = 0.93; C_methylene_—H = 0.97 Å, N—H = 0.86 Å; *U*_iso_(H) = 1.2 *U*_eq_(carrier atom). The disorder was treated with the following constraints and restraints: The anisotropic displacement parameters of the pairs of the atoms C11, C11' and C12, C12' were set equal by the command EADP (Sheldrick, 2008). The corresponding interatomic distances C11—C12 and C11'-C12' were restrained to 1.485 (10) Å while the distances C10—C11, C10—C11', C12—C13, C12'-C13 were restrained to 1.520 (10) Å. (The values of these distances were excerpted from the Cambridge Crystal Structure Database (Allen, 2002) for the structures that contained the similar fragment –N?cyclopentane as it is contained in the title structure. The searched structures were without disorder, errors and with *R*-factor < 0.05. 4 structures with the following REFCODES were found: HULJON, KERWUA, NAQSAZ and RAKHUH.) In addition, for the disordered parts the restrain SAME has been applied (Müller *et al.*, 2006; Sheldrick, 2008). The respective occupancies were refined to 0.628 (9) and to 0.372 (9).

### Crystal data

**Table:**

C_13_H_15_N_5_O	*F*_000_ = 544
*M**_r_* = 257.30	*D*_x_ = 1.300 Mg m^−^^3^
Monoclinic, *P*2_1_/*c*	Melting point = 490–492 K
Hall symbol: -P 2ybc	Mo *K*α radiation λ = 0.71073 Å
*a* = 11.926 (3) Å	Cell parameters from 526 reflections
*b* = 9.126 (2) Å	θ = 2.8–19.1º
*c* = 12.095 (3) Å	µ = 0.09 mm^−^^1^
β = 93.174 (5)º	*T* = 295 (2) K
*V* = 1314.4 (5) Å^3^	Block, colorless
*Z* = 4	0.32 × 0.24 × 0.11 mm

### Data collection

**Table:**

Bruker SMART CCD area-detector diffractometer	2323 independent reflections
Radiation source: fine-focus sealed tube	1114 reflections with *I* > 2σ(*I*)
Monochromator: graphite	*R*_int_ = 0.065
*T* = 295(2) K	θ_max_ = 25.1º
φ and ω scans	θ_min_ = 1.7º
Absorption correction: multi-scan(SADABS; Sheldrick, 1996)	*h* = −14→14
*T*_min_ = 0.972, *T*_max_ = 0.990	*k* = −10→10
6723 measured reflections	*l* = −8→14

### Refinement

**Table:**

Refinement on *F*^2^	Primary atom site location: structure-invariant direct methods
Least-squares matrix: full	Secondary atom site location: difference Fourier map
*R*[*F*^2^ > 2σ(*F*^2^)] = 0.060	Hydrogen site location: inferred from neighbouring sites
*wR*(*F*^2^) = 0.170	H-atom parameters constrained
*S* = 1.01	*w* = 1/[σ^2^(*F*_o_^2^) + (0.071*P*)^2^] where *P* = (*F*_o_^2^ + 2*F*_c_^2^)/3
2323 reflections	(Δ/σ)_max_ < 0.001
173 parameters	Δρ_max_ = 0.17 e Å^−^^3^
7 restraints	Δρ_min_ = −0.26 e Å^−^^3^
74 constraints	Extinction correction: none

### Special details

**Table:**

Geometry. All e.s.d.'s (except the e.s.d. in the dihedral angle between two l.s. planes) are estimated using the full covariance matrix. The cell e.s.d.'s are taken into account individually in the estimation of e.s.d.'s in distances, angles and torsion angles; correlations between e.s.d.'s in cell parameters are only used when they are defined by crystal symmetry. An approximate (isotropic) treatment of cell e.s.d.'s is used for estimating e.s.d.'s involving l.s. planes.
Refinement. Refinement of *F*^2^ against ALL reflections. The weighted *R*-factor *wR* and goodness of fit *S* are based on *F*^2^, conventional *R*-factors *R* are based on *F*, with *F* set to zero for negative *F*^2^. The threshold expression of *F*^2^ > σ(*F*^2^) is used only for calculating *R*-factors(gt) *etc*. and is not relevant to the choice of reflections for refinement. *R*-factors based on *F*^2^ are statistically about twice as large as those based on *F*, and *R*- factors based on ALL data will be even larger.

### Fractional atomic coordinates and isotropic or equivalent isotropic displacement parameters (Å^2^)

**Table:**

	*x*	*y*	*z*	*U*_iso_*/*U*_eq_	Occ. (<1)
O1	0.45566 (18)	−0.0903 (2)	0.2935 (2)	0.0720 (8)	
N1	0.2040 (3)	0.0346 (3)	0.4948 (3)	0.0758 (10)	
N2	0.3131 (3)	0.0413 (3)	0.4947 (2)	0.0687 (9)	
N3	0.3419 (2)	0.1243 (3)	0.4072 (2)	0.0525 (7)	
N4	0.55577 (19)	0.0916 (3)	0.2198 (2)	0.0533 (8)	
H4	0.5828	0.1785	0.2287	0.064*	
N5	0.5824 (2)	0.0084 (3)	0.1280 (2)	0.0589 (8)	
C1	0.2485 (3)	0.1717 (3)	0.3495 (3)	0.0502 (9)	
C2	0.1608 (3)	0.1146 (4)	0.4060 (3)	0.0603 (10)	
C3	0.0492 (3)	0.1441 (5)	0.3729 (4)	0.0807 (12)	
H3	−0.0103	0.1054	0.4099	0.097*	
C4	0.0325 (3)	0.2335 (5)	0.2823 (4)	0.0862 (13)	
H4A	−0.0407	0.2569	0.2579	0.103*	
C5	0.1217 (3)	0.2910 (4)	0.2253 (3)	0.0779 (12)	
H5	0.1061	0.3511	0.1643	0.094*	
C6	0.2317 (3)	0.2607 (4)	0.2572 (3)	0.0613 (10)	
H6	0.2912	0.2978	0.2192	0.074*	
C7	0.4582 (2)	0.1429 (3)	0.3833 (3)	0.0534 (9)	
H7A	0.5055	0.1261	0.4499	0.064*	
H7B	0.4706	0.2425	0.3587	0.064*	
C8	0.4891 (2)	0.0361 (3)	0.2940 (3)	0.0493 (8)	
C9	0.6663 (3)	0.0541 (3)	0.0777 (3)	0.0529 (9)	
C10	0.7449 (3)	0.1752 (4)	0.1090 (3)	0.0871 (13)	
H10A	0.7701	0.1683	0.1865	0.104*	
H10B	0.7094	0.2697	0.0959	0.104*	
C13	0.7025 (3)	−0.0176 (4)	−0.0264 (3)	0.0703 (11)	
H13A	0.7280	−0.1170	−0.0116	0.084*	
H13B	0.6409	−0.0203	−0.0823	0.084*	
C11	0.8426 (3)	0.1542 (4)	0.0347 (3)	0.116 (2)	0.628 (9)
H11A	0.8725	0.2479	0.0126	0.139*	0.628 (9)
H11B	0.9022	0.0982	0.0725	0.139*	0.628 (9)
C12	0.7939 (3)	0.0729 (4)	−0.0628 (3)	0.122 (4)	0.628 (9)
H12A	0.7659	0.1412	−0.1193	0.147*	0.628 (9)
H12B	0.8508	0.0119	−0.0939	0.147*	0.628 (9)
C11'	0.8241 (12)	0.1710 (16)	0.0129 (13)	0.116 (2)	0.372 (9)
H11C	0.7958	0.2337	−0.0471	0.139*	0.372 (9)
H11D	0.8985	0.2044	0.0376	0.139*	0.372 (9)
C12'	0.8279 (9)	0.0159 (18)	−0.0244 (18)	0.122 (4)	0.372 (9)
H12C	0.8707	−0.0454	0.0280	0.147*	0.372 (9)
H12D	0.8576	0.0068	−0.0971	0.147*	0.372 (9)

### Atomic displacement parameters (Å^2^)

**Table:**

	*U*^11^	*U*^22^	*U*^33^	*U*^12^	*U*^13^	*U*^23^
O1	0.0847 (17)	0.0349 (13)	0.100 (2)	−0.0061 (12)	0.0413 (15)	−0.0016 (12)
N1	0.081 (2)	0.061 (2)	0.090 (3)	0.0051 (17)	0.037 (2)	0.0106 (19)
N2	0.080 (2)	0.0599 (19)	0.068 (2)	0.0112 (16)	0.0267 (17)	0.0135 (16)
N3	0.0573 (17)	0.0441 (16)	0.0577 (18)	0.0037 (14)	0.0164 (15)	0.0029 (14)
N4	0.0522 (16)	0.0376 (15)	0.072 (2)	−0.0079 (12)	0.0153 (15)	−0.0014 (14)
N5	0.0638 (19)	0.0484 (17)	0.0662 (19)	−0.0058 (14)	0.0187 (16)	−0.0038 (15)
C1	0.050 (2)	0.048 (2)	0.054 (2)	0.0047 (16)	0.0128 (18)	−0.0080 (19)
C2	0.059 (2)	0.053 (2)	0.071 (3)	0.0009 (19)	0.023 (2)	−0.009 (2)
C3	0.061 (3)	0.089 (3)	0.095 (3)	−0.009 (2)	0.029 (2)	−0.020 (3)
C4	0.050 (2)	0.123 (4)	0.086 (3)	0.003 (2)	0.006 (2)	−0.021 (3)
C5	0.071 (3)	0.096 (3)	0.066 (3)	0.014 (2)	0.005 (2)	0.003 (2)
C6	0.060 (2)	0.068 (2)	0.056 (2)	0.0048 (19)	0.0079 (19)	−0.002 (2)
C7	0.053 (2)	0.0397 (18)	0.069 (2)	0.0004 (16)	0.0123 (17)	0.0030 (18)
C8	0.0459 (19)	0.0354 (18)	0.068 (2)	0.0037 (15)	0.0118 (17)	0.0063 (17)
C9	0.051 (2)	0.048 (2)	0.061 (2)	0.0030 (16)	0.0114 (18)	0.0065 (18)
C10	0.075 (3)	0.073 (3)	0.117 (4)	−0.026 (2)	0.042 (3)	−0.015 (2)
C13	0.069 (2)	0.073 (3)	0.071 (3)	−0.001 (2)	0.015 (2)	−0.004 (2)
C11	0.090 (4)	0.104 (4)	0.159 (6)	−0.037 (3)	0.068 (4)	−0.032 (4)
C12	0.105 (5)	0.148 (9)	0.119 (8)	−0.039 (5)	0.058 (5)	−0.038 (7)
C11'	0.090 (4)	0.104 (4)	0.159 (6)	−0.037 (3)	0.068 (4)	−0.032 (4)
C12'	0.105 (5)	0.148 (9)	0.119 (8)	−0.039 (5)	0.058 (5)	−0.038 (7)

### Geometric parameters (Å, °)

**Table:**

O1—C8	1.220 (3)	C7—H7B	0.9700
N1—N2	1.302 (4)	C9—C10	1.484 (4)
N1—C2	1.375 (4)	C9—C13	1.503 (4)
N2—N3	1.361 (3)	C10—C11	1.522 (4)
N3—C1	1.352 (4)	C10—C11'	1.538 (8)
N3—C7	1.443 (3)	C10—H10A	0.9700
N4—C8	1.332 (3)	C10—H10B	0.9700
N4—N5	1.397 (3)	C13—C12	1.4555
N4—H4	0.8604	C13—C12'	1.526 (9)
N5—C9	1.270 (4)	C13—H13A	0.9700
C1—C2	1.383 (4)	C13—H13B	0.9700
C1—C6	1.387 (4)	C11—C12	1.4841
C2—C3	1.395 (5)	C11—H11A	0.9700
C3—C4	1.372 (5)	C11—H11B	0.9700
C3—H3	0.9300	C12—H12A	0.9700
C4—C5	1.401 (5)	C12—H12B	0.9700
C4—H4A	0.9300	C11'—C12'	1.487 (9)
C5—C6	1.374 (5)	C11'—H11C	0.9700
C5—H5	0.9300	C11'—H11D	0.9700
C6—H6	0.9300	C12'—H12C	0.9700
C7—C8	1.516 (4)	C12'—H12D	0.9700
C7—H7A	0.9700		
			
N2—N1—C2	107.8 (3)	C9—C10—C11'	101.2 (6)
N1—N2—N3	108.8 (3)	C9—C10—H10A	110.9
C1—N3—N2	110.1 (3)	C11—C10—H10A	110.9
C1—N3—C7	129.2 (3)	C11'—C10—H10A	123.9
N2—N3—C7	120.6 (3)	C9—C10—H10B	110.9
C8—N4—N5	120.0 (3)	C11—C10—H10B	110.9
C8—N4—H4	120.0	C11'—C10—H10B	100.3
N5—N4—H4	120.0	H10A—C10—H10B	108.9
C9—N5—N4	115.0 (3)	C12—C13—C9	105.08 (18)
N3—C1—C2	104.4 (3)	C9—C13—C12'	103.0 (7)
N3—C1—C6	133.0 (3)	C12—C13—H13A	110.7
C2—C1—C6	122.6 (3)	C9—C13—H13A	110.7
N1—C2—C1	109.0 (3)	C12'—C13—H13A	83.5
N1—C2—C3	129.6 (4)	C12—C13—H13B	110.7
C1—C2—C3	121.4 (4)	C9—C13—H13B	110.7
C4—C3—C2	116.0 (4)	C12'—C13—H13B	136.0
C4—C3—H3	122.0	H13A—C13—H13B	108.8
C2—C3—H3	122.0	C12—C11—C10	104.7 (2)
C3—C4—C5	122.3 (4)	C12—C11—H11A	110.8
C3—C4—H4A	118.9	C10—C11—H11A	111.0
C5—C4—H4A	118.9	C12—C11—H11B	110.8
C6—C5—C4	121.7 (4)	C10—C11—H11B	110.7
C6—C5—H5	119.1	H11A—C11—H11B	108.9
C4—C5—H5	119.1	C13—C12—C11	108.1
C5—C6—C1	115.9 (3)	C13—C12—H12A	110.1
C5—C6—H6	122.0	C11—C12—H12A	110.1
C1—C6—H6	122.0	C13—C12—H12B	110.1
N3—C7—C8	110.0 (2)	C11—C12—H12B	110.1
N3—C7—H7A	109.7	H12A—C12—H12B	108.4
C8—C7—H7A	109.7	C12'—C11'—C10	106.4 (10)
N3—C7—H7B	109.7	C12'—C11'—H11C	110.4
C8—C7—H7B	109.7	C10—C11'—H11C	110.4
H7A—C7—H7B	108.2	C12'—C11'—H11D	110.4
O1—C8—N4	124.3 (3)	C10—C11'—H11D	110.4
O1—C8—C7	121.3 (3)	H11C—C11'—H11D	108.6
N4—C8—C7	114.4 (3)	C11'—C12'—C13	98.6 (10)
N5—C9—C10	128.7 (3)	C11'—C12'—H12C	112.1
N5—C9—C13	121.9 (3)	C11'—C12'—H12D	112.1
C10—C9—C13	109.4 (3)	C13—C12'—H12D	112.1
C9—C10—C11	104.4 (3)		

### Hydrogen-bond geometry (Å, °)

**Table:**

*D*—H···*A*	*D*—H	H···*A*	*D*···*A*	*D*—H···*A*
N4—H4···O1^i^	0.86	2.17	2.910 (3)	144
C7—H7B···O1^i^	0.97	2.58	3.435 (4)	147
C7—H7B···N5^i^	0.97	2.51	3.372 (4)	147
C13—H13A···Cg1^ii^	0.97	2.79	3.729 (4)	163
C12'—H12C···Cg2^ii^	0.97	2.99	3.820 (19)	145

Symmetry codes: (i) −*x*+1, *y*+1/2, −*z*+1/2; (ii) −*x*+1, *y*−1/2, −*z*+1/2.
